# Gelation and the Self-Healing Behavior of the Chitosan–Catechol Hydrogel

**DOI:** 10.3390/polym14214614

**Published:** 2022-10-30

**Authors:** Yu-Ting Lan, Qian-Pu Cheng, Junpeng Xu, Shih-Ho Lin, Jhih-Min Lin, Shan-hui Hsu

**Affiliations:** 1 Institute of Polymer Science and Engineering, National Taiwan University, Taipei 10617, Taiwan; 2 National Synchrotron Radiation Research Center, Hsinchu 30076, Taiwan; 3 Institute of Cellular and System Medicine, National Health Research Institutes, Miaoli 35053, Taiwan

**Keywords:** chitosan–catechol, self-healing hydrogel, gelation, network structure, adhesive, scaffold

## Abstract

Mussel-inspired adhesive hydrogels have been developed in biomedical fields due to their strong adhesive property, cohesive capability, biocompatibility, and hemostatic ability. Catechol-functionalized chitosan is a potential polymer used to prepare adhesive hydrogels. However, the unique gelation mechanism and self-healing properties of catechol-grafted chitosan alone have not yet been explored. Herein, catechol-grafted chitosan (CC) was synthesized and further concentrated to obtain the self-healing CC hydrogels. The gelation mechanism of CC hydrogels may be attributed to the formation of hydrogen bonding, cation–π interactions, Michael addition, or Schiff base reactions during concentration phases. Rheological studies showed that the CC hydrogel owned self-healing properties in repeated damage–healing cycles. Coherent small-angle X-ray scattering (SAXS) analyses revealed the formation of a mesoscale structure (~9 nm) as the solid content of the hydrogel increased. In situ SAXS combined with rheometry verified the strain-dependent behavior of the CC hydrogel. The CC hydrogel displayed the osmotic-responsive behavior and enhanced adhesive strength (0.38 N/cm^2^) after immersion in the physiological saline. The CC scaffold prepared by lyophilizing the CC hydrogel revealed a macroporous structure (~200 µm), a high swelling ratio (9656%), good compressibility, and durability. This work provides an insight into the design of using chitosan–catechol alone to produce hydrogels or scaffolds with tunable mechanical properties for further applications in biomedical fields.

## 1. Introduction

Chitosan is a natural polysaccharide that can be obtained by the deacetylation of chitin [1]. It has been widely used in biomedical and environmental fields due to its biodegradable, nontoxic, and biocompatible properties [2]. Although chitosan has many advantages, some applications are limited, owing to its insolubility in water [3]. Oligochitosan is water-soluble but has a rather low molecular weight (<10 kDa) [4]. Meanwhile, water-soluble chitosan derivatives can be prepared through chemical modification methods to overcome the insolubility of chitosan. Glycol chitosan is one common water-soluble chitosan derivative and is prepared by introducing the hydrophilic glycol groups onto chitosan [5]. Other water-soluble chitosan derivatives include zwitterionic chitosans such as carboxyethyl chitosan (CEC) and carboxymethyl chitosan (CMC) which are synthesized, respectively, by the N,O-acetylation and O-carboxymethylation of chitosan [6,7]. Moreover, catechol-conjugated chitosan can be prepared using 3,4-dihydroxyhydrocinnamic acid to conjugate catechol groups onto chitosan to achieve water solubility [8]. These water-soluble chitosan derivatives are important biopolymers because they are more convenient for biomedical applications.

Hydrogels are three-dimensional network of hydrophilic polymers crosslinked by intermolecular or intramolecular forces, which can absorb a large amount of liquid fluids and easily swell in water without dissolving [9]. They mimic the extracellular matrix of biological tissues because of their unique physicochemical properties [10]. Chitosan-based hydrogels are attractive biomaterials owing to their versatility, biodegradability, biocompatibility, and low cost [11]. Chitosan-based hydrogels are often prepared through the reaction of chitosan with different crosslinkers, and among which glutaraldehyde is a conventional crosslinker [12]. The chitosan-based hydrogels may further own self-healing properties if a dynamic Schiff base crosslinker is employed [13]. Meanwhile, adhesive chitosan-based hydrogels can also be prepared from catechol-conjugated glycol chitosan after dissolution in the buffer solution of pH 7.4 [14]. Such a method to prepare chitosan hydrogels can avoid the use of potentially toxic crosslinkers and thus is safer for biomedical applications.

Catechols are universally present in many natural systems, where a typical example is the family of mussel adhesive proteins. 3,4-Dihydroxy-L-phenylalanine (DOPA) is the main component of mussel adhesive proteins, and catechol is the side chain of DOPA, which can exhibit excellent adhesion to various organic/inorganic surfaces [15]. The adhesion observed in DOPA- and Lys-rich mussel adhesive proteins is mainly due to cation–π interactions [16]. Cation–π interactions promote the cohesion of materials rich in cationic and aromatic functional groups [17]. Hydrogen bonding can also contribute to the coacervation of the polymer [18]. Moreover, catechols are readily oxidized in air or under oxidative conditions to form reactive *o*-quinones to further form covalent crosslinks [19]. *O*-quinones can react with amines via a Schiff base reaction or Michael addition [20]. Furthermore, *o*-quinones can also form catechol–catechol crosslinks with non-oxidized catechols through the dismutation reaction [21]. These synergistic interactions can increase the cohesion of the polymer chains to enhance the gelation [17]. Because of the special properties and crosslinking ability, catechol has been introduced to a few polymer chains to produce polymer–catechol, including polyethylene glycol–catechol [22], hyaluronic acid–catechol [23], alginate–catechol [24], and chitosan–catechol [25]. The chitosan–catechol can form a hydrogel network through the addition of crosslinker [26], coordination with metal ions [27], or spontaneous crosslinking of oxidized catechol with amine through Schiff base reactions or Michael addition [14].

Although catechol-grafted chitosan is water-soluble under neutral conditions (pH < 7) and can form a thin adhesive layer hydrogel on the surface of the substrate, its water solubility is very limited (only about 0.05%) in the weak base (pH > 7.5) [8]. The gelation behavior and self-healing properties of catechol-grafted chitosan have not been examined so far. In the present study, we prepared chitosan–catechol (CC) in water and concentrated to obtain CC self-healing hydrogels, where the gelation mechanism was explored. During the concentration process, as the distances between the polymer chains gradually decreases, the kinetics of the crosslinking reaction of catechol may increase to promote gelation. The stability and self-healing properties of the CC hydrogels were analyzed by rheological studies, while the microstructure changes of the CC hydrogels were explored using SAXS and in situ SAXS. The osmotic response and adhesive function of the hydrogel, as well as the compressibility of the CC scaffold, were verified.

## 2. Materials and Methods

### 2.1. Materials

Chitosan (MW ∼120 kDa, degree of deacetylation 76%), 2-(*N*-morpholino) ethanesulfonic acid hydrate (MES), hydrocaffeic acid (HCA, 3,4-dihydroxyhydrocinnamic acid), N-(3-dimethylaminopropyl)-N′-ethylcarbodiimide hydrochloride (EDC), N-hydroxysuccinimide (NHS), and NaOH were purchased from Sigma. Hydrochloric acid (35%) was purchased from Showa. Low-glucose Dulbecco’s modified Eagle’s medium (LG-DMEM) was purchased from Gibco. All materials were applied in commercial form.

### 2.2. Synthesis and Characterization of Chitosan–Catechol (CC)

CC was synthesized according to the previous literature [28], with slight changes. Chitosan (200 mg) was dissolved in 2.0 mL of 1 N hydrochloric acid and then added 8.0 mL of MES buffer (50 mM) to dilute the solution. Subsequently, hydrocaffeic acid (0.6 mmol, 109.3 mg), EDC (0.6 mmol, 115.0 mg), and NHS (0.6 mmol, 69.2 mg) were added to 90 mL of MES buffer and then uniformly mixed with the above solution, followed by adjusting the pH to 5.5 with 1 N NaOH. The mixture was reacted at 25 °C in dark under stirring. After 24 h, the solution was dialyzed using a dialysis membrane (MWCO 12−14 kDa) against pH 5.0 HCl solution for 2 days and deionized water for 4 h to obtain the purified aqueous solution of CC (about 0.2 wt%, pH 6). The solution was filtered using suction filtration and kept in a refrigerator before use.

Proton nuclear magnetic resonance (^1^H NMR) spectroscopy was used to identify CC. For preparation of the samples, a purified CC solution was first freeze-dried to obtain the solid CC. Subsequently, the solution of CC (10 mg/mL) in D_2_O and the solution of chitosan (10 mg/mL) in CH_3_COOD/D_2_O (2% *v*/*v*) were measured by an NMR spectrometer (AVIII-500 MHz FT-NMR, Bruker, Billerica, MA, USA). The substitution of catechol groups in CC was quantified by UV–vis spectroscopy. CC solution (1.5 mg/mL in deionized water) and chitosan solution (1.5 mg/mL in 0.01 N HCl) were scanned at wavelengths from 230 nm to 500 nm using a UV–vis spectrometer (SpectraMax iD3, Molecular Devices, San Jose, CA, USA). The calibration curve of standard hydrocaffeic acid solutions at a wavelength of 280 nm was used to calculate the catechol content in CC.

### 2.3. Preparation of CC Hydrogels

CC hydrogels with different concentrations were prepared using the rotary concentration method. The aforementioned solution of CC was concentrated to 0.5%, 1%, 1.5%, and 2%, respectively, using a rotary evaporator (Rotavapor R-100, BUCHI, Switzerland) with temperature maintained at 25 °C. The CC hydrogels were allowed to form from the concentrated solutions at room temperature after placed for 3 days.

### 2.4. Rheological Properties of CC Hydrogels

The rheological properties of CC hydrogels were evaluated using a rheometer (HR-2, TA Instruments, Newcastle, DE, USA) with a diameter of 20 mm parallel plate geometry at 25 °C. The storage shear modulus and loss shear modulus (G′ and G′′) of the hydrogels were measured in the range of 0.1 to 10 Hz frequency (frequency sweep mode) at a constant strain of 1% or 2%. The dynamic strain sweep was investigated from 0.1% to 1500% strain at a frequency of 1 Hz. The damage–healing cycles at the alternate higher strain (1100%) and lower strain (1%) at a constant frequency of 1 Hz were recorded to assess the self-healing properties of CC hydrogels. The shear-thinning behavior of the CC hydrogel was evaluated by the steady shear experiment, where the steady shear viscosity was determined in a shear rate ranging from 0.1 to 1000 s^−1^.

### 2.5. Coherent SAXS and In Situ SAXS

The microstructure of CC hydrogels with different solid contents was investigated by coherent SAXS at the beamline 25A1 of Taiwan Photon Source (TPS 25A1) at National Synchrotron Radiation Research Center (NSRRC) in Hsinchu, Taiwan. Moreover, the microstructure change during the deformation of the hydrogel was investigated by in situ SAXS equipped with a double-cylinder rheometer (Physica MCR-501, Anton Paar, Graz, Austria). The photon energy of both was set to 8 keV. For SAXS, samples were detected at different times (0 min, 15 min, and 30 min) after loading to the sample holder. The scattering vector (q) ranged from 0.0027 to 0.28 Å^−1^. For in situ SAXS, the sample was loaded in the double-cylinder tool (inner diameter 20.5 mm and outer diameter 21 mm) and measured under several oscillatory strains by the rheometer. The scattering vector (q) ranged from 0.0023 to 0.28 Å^−1^.

### 2.6. Macroscopic Self-Healing Property and Injectability of the CC Hydrogel 

For a visual observation of the self-healing phenomenon of the hydrogel, two hydrogel discs were prepared and cut into two halves. One hydrogel disc was dyed with the trypan blue to make the color different. The two semicircular hydrogels with different colors were put together for a certain period of time. The healing ability of the two hydrogel pieces was evaluated by pulling the whole hydrogel to failure with tweezers. The injectability was assessed by injecting the hydrogel through a 30-gauge syringe needle (i.e., 160 µm internal diameter).

### 2.7. Osmotic-Responsive Properties of the CC Hydrogel

The CC hydrogel was immersed in the physiological saline (0.9% NaCl solution) or culture medium (LG-DMEM) to evaluate the osmotic response of the hydrogel. The swelling kinetics of the hydrogel in saline or culture medium were studied by measuring weight changes in the hydrogel for a given time at 25 °C. After immersion for a specific time interval, the hydrogel was taken out and weighed after the liquid on the surface was removed. The water retention ratio (WR) was described as the percentage of water content of the hydrogel after immersion for a certain time relative to the water content of the initial hydrogel. The data were recorded in triplicate. The WR was calculated as the Equation (1):

(1) $$\mathrm{WR}=\left(W_{t}-W_{d}\right)/\left(W_{i}-W_{d}\right)\times 100\%$$

where *W_t_* is the weight of the hydrogel at a given time during swelling or deswelling, *W_d_* is the weight of the dry hydrogel, and *W_i_* is the weight of the initial hydrogel before soaking. The deswelling process of the hydrogel was quantitatively analyzed by a first-ordered kinetics with the following Equation (2) [29]:

(2) $$\ln\;\left[\left(W_{t}-W_{e}\right)/\left(W_{i}-W_{e}\right)\right]=-kt$$

where *W_e_* is the weight of the hydrogel in the equilibrium deswelling state, *t* is the deswelling time, and *k* is a constant used to describe the deswelling rate.

The rigidity of the hydrogel after immersion was evaluated by the rheometer using 20 mm parallel plate geometry at 25 °C. The storage shear modulus and loss shear modulus (G′ and G′′) of the hydrogel were measured in the range of 0.1 to 10 Hz frequency (frequency sweep mode) at a constant strain of 1%.

### 2.8. Adhesive Properties of CC Hydrogels

The weight-bearing experiment, according to the previous literature [28], was used to determine the adhesive properties of the pristine CC hydrogel and the de-swollen one in saline. Two glass slides (2 cm × 2 cm × 0.2 cm) attached with the commercial artificial skins (Hartmann) and weight sets (100, 50, 20, and 10 g) were used to prepare the weight-bearing device. The hydrogel was loaded between the two glass slides, whereby the artificial skin sides were contacted to the hydrogel and set for 10 min at room temperature before the measurement. The binding strength of the hydrogel was calculated as the maximum load divided by the contacted area.

### 2.9. Preparation and Characterization of CC Scaffolds

CC scaffolds were produced via the lyophilization of equilibrium CC hydrogels. The preparation process of CC hydrogels was mentioned above. To make scaffolds, CC hydrogels were kept at −20 °C for 24 h and then freeze-dried for 24 h. Scanning electron microscope (SEM, Hitachi TM 3000, Tokyo, Japan) was used to observe the porous structures in the cross-section of the CC scaffolds by applying a voltage of 3 kV. The porosity of the CC scaffolds was assessed by immersing the scaffolds in water at room temperature. The data were recorded in triplicate. The value of the porosity was calculated by the following Equation (3):

(3) $$\mathrm{Porosity}=\left(W_{w}-W_{d}\right)/\rho V\times 100\%$$

where *W_w_* is the wet weight of the CC scaffold after immersion in water, *W_d_* is the weight of the initial dry CC scaffold, *ρ* is the density of water, and *V* is the volume of the CC scaffold.

The swelling ratio of the CC scaffolds was measured at room temperature. The dried CC scaffolds were weighed (*W_d_*) and then immersed in water. After swelling, the swollen scaffolds were weighed (*W_w_*) after removing the redundant water. Data of the swelling ratio were recorded in triplicate. The swelling ratio of the scaffolds was calculated as the Equation (4):

(4) $$\mathrm{Swelling}\;\mathrm{ratio}=\left(W_{w}-W_{d}\right)/W_{d}\times 100\%$$

The dynamic compressive modulus of the hydrated CC scaffolds was evaluated by the dynamic mechanical analyzer (DMA, Q800, TA Instruments, Newcastle, DE, USA) at 1% compressive strain and a constant frequency of 1 Hz at 25 °C.

### 2.10. Statistical Analysis

Statistical analysis was performed by the Student *t*-test. The quantitative data in the current study were presented as mean ± standard deviation. The experimental data with the values of *p* < 0.05 were considered statistically significant.

## 3. Results

### 3.1. Synthesis and Characterization of CC

**Figure 1:**
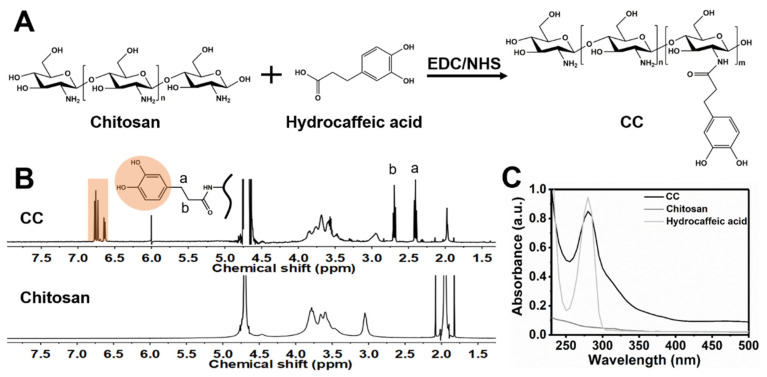
Synthesis and characterization of chitosan–catechol (CC). (**A**) The synthetic procedure of CC using EDC/NHS chemistry to conjugate hydrocaffeic acid with chitosan. (**B**) ^1^H NMR spectra of CC and chitosan. (**C**) UV–vis spectra of CC solution, chitosan solution, and hydrocaffeic acid solution in the range of 200 nm to 500 nm.

CC was synthesized via EDC/NHS chemistry to conjugate the catechol groups to the chitosan backbone by forming an amine bond between the primary amine group in chitosan and the carboxylic acid group in hydrocaffeic acid (Figure 1A). The chemical structures of chitosan and CC were confirmed by ^1^H NMR spectroscopy (Figure 1B). The signals of the catechol ring and methylene protons at δ 6.63–6.77 ppm and δ 2.41, 2.69 ppm (H_a_ and H_b_), respectively, verified the successful conjugation of catechol groups to the chitosan. In UV–vis spectra, the hydrocaffeic acid and CC showed the maximum absorbance values at the wavelength of 280 nm, whereas chitosan displayed no absorbance value at 280 nm, indicating that catechol groups were effectively conjugated to chitosan (Figure 1C). In addition, there was no peak for CC at wavelengths longer than 300 nm, representing that the conjugated catechol was not oxidized to quinone during the preparation of CC [23]. The degree of catechol substitution in CC was approximately 12.6%, based on the calibration curve generated from the standard hydrocaffeic acid solutions at the absorption intensity of 280 nm (Figure S1, Supplementary Material).

### 3.2. Characterization of CC Hydrogels

**Figure 2:**
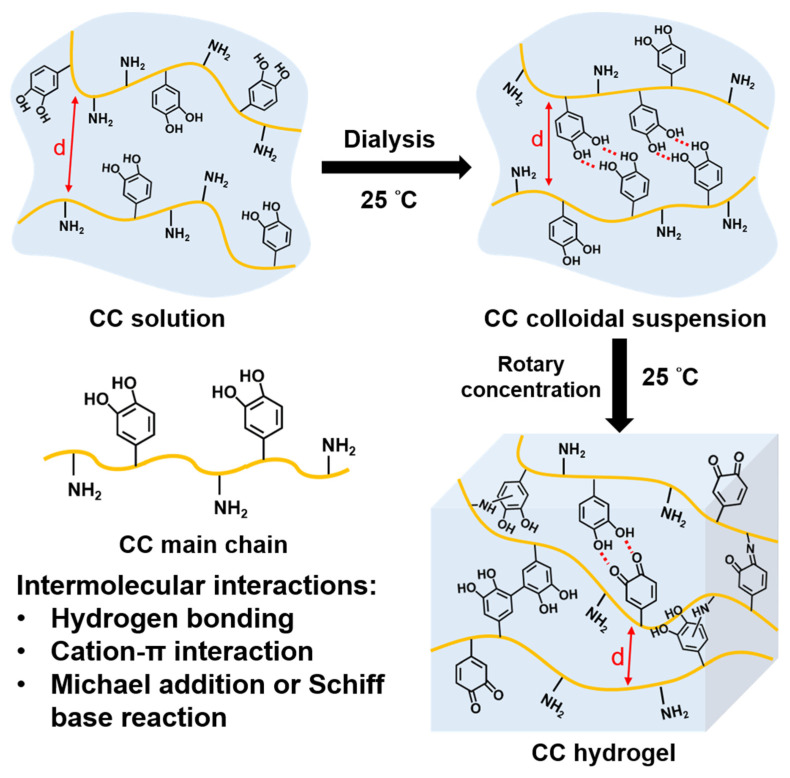
A scheme for the hypothetical mechanism of forming CC colloidal suspension during the dialysis process and forming the CC hydrogel during the rotary concentration process in the current study.

The CC colloidal suspension in the study formed during the dialysis process, while CC hydrogels formed after concentration to higher solid contents at 25 °C. The hypothetical scheme for the structure formation is illustrated in Figure 2. In the initial CC solution, the distances of CC main chains are far enough to prevent the crosslink. During the dialysis process, parts of the CC main chains become closer and form hydrogen bonds to produce the colloidal suspension. During concentration, the CC main chains become even closer, which promotes gelation due to the accelerated crosslinking kinetics of catechol. The intermolecular interactions in CC hydrogels are mainly composed of hydrogen bonding, cation–π interactions, and Schiff base reactions or Michael addition caused by the reaction of oxidized catechols with amines.

**Figure 3:**
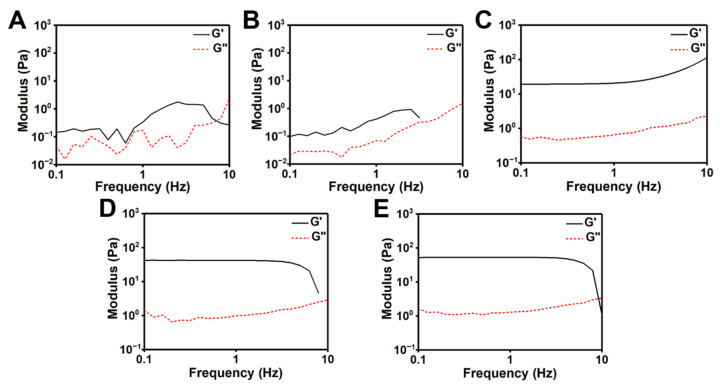
Rheological properties of various CC hydrogels, with solid contents of (**A**) 0.2%, (**B**) 0.5%, (**C**) 1%, (**D**) 1.5%, and (**E**) 2% at 25 °C. The storage modulus (G′) and loss modulus (G′′) were measured by the frequency sweep.

The rheology of various CC hydrogels was verified by frequency sweep experiments. For the fluid-like CC suspensions with solid contents of 0.2% and 0.5% [Figure 3A,B], the shear storage modulus (G′) was greater than the shear loss modulus (G′′), but the G′ had unstable low values (0.3 Pa for 0.2% and 0.4 Pa for 0.5%), typical of colloidal suspensions. For the hydrogel at the solid content of 1% (Figure 3C), G′ had stable values with an upward trend in higher frequencies (1–10 Hz), indicating that it was a weak hydrogel. For the hydrogel with the solid contents of 1.5% and 2% (Figure 3D and E), G′ remained invariant under a broad range of frequencies (0.1–7 Hz) except for the sudden drop at the higher frequency (~7 Hz). The storage moduli (at 1 Hz) and abbreviated names of CC suspensions/hydrogels with different solid contents are listed in Table 1. The moduli only differed slightly between hydrogels with solid contents of 1.5% (i.e., the CC_1.5_ hydrogel) and 2% (i.e., the CC_2.0_ hydrogel).

**Figure 4:**
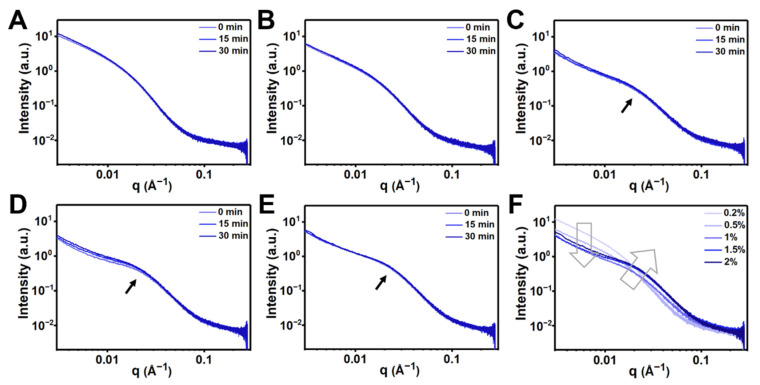
Coherent SAXS profiles for CC hydrogels with different solid contents at 25 °C in different scales of setting time (0, 15, and 30 min). The solid contents of CC hydrogels were (**A**) 0.2%, (**B**) 0.5%, (**C**) 1%, (**D**) 1.5%, and (**E**) 2%. (**F**) Comparison of the scattering profiles among CC hydrogels with different solid contents (data obtained at 30 min).

Microstructures of CC hydrogels at different solid contents were explored using coherent SAXS. The scattering curves of the hydrogels after loading to the sample holder for different times are shown in Figure 4A–E. The time at the sample loading was set as *t* = 0, and the scattering intensities were collected after 0 min, 15 min, and 30 min, respectively. For all hydrogels except CC_1.5_, the intensities of each sample were almost the same for different setting times, indicating that the structures of the hydrogels were stable and did not significantly change with time. For CC_1.5_, the intensities were slightly increased as the time increased, demonstrating that the structure of the hydrogel was critical, more sensitive to the strain changes, and required a longer time to return to the steady state. Figure 4F shows a comparison of the scattering curves among CC hydrogels with different solid contents at the steady state (i.e., data collected at 30 min). At the q range < 0.01 Å^−1^, CC_0.2_ had the maximum intensity and then decreased as the solid content of the hydrogels increased, which revealed that the uniformly dispersed chain structure in a large scale was in the form of colloidal suspension. Moreover, the increased solid contents in the CC_2.0_ hydrogel may contribute to the greater scattering intensity at the high-q range, resulting in an up-shift tendency in the curve. As the solid content of the hydrogel increased, a shoulder at q ~ 0.02–0.04 Å^−1^ appeared and became more obvious, indicating the gradual formation of a featured mesoscale structure (cluster size ~ 9 nm). This structure represented the formation of clusters in the hydrogel network. Based on the rheological evaluation and coherent SAXS investigation, the CC_1.5_ hydrogel was selected for further study.

### 3.3. Strain-Dependent Performance of the CC Hydrogel

**Figure 5:**
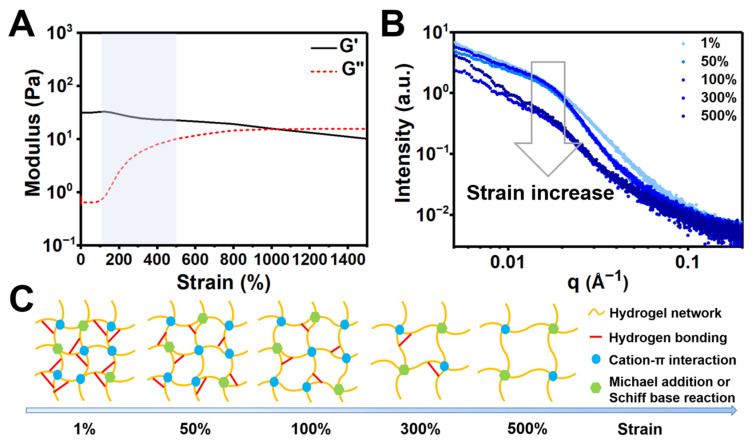
Strain-dependent and in situ SAXS for the CC hydrogel at 25 °C (solid content 1.5%). (**A**) The G′ and G′′ values by strain sweep from 0.1% to 1500% dynamic strain at the frequency of 1 Hz. (**B**) In situ SAXS profiles of the hydrogel upon increasing strains. (**C**) A scheme for the possible network changes in the CC hydrogel as the strain increases.

In situ SAXS analysis combined with rheometry was used to evaluate the strain-dependent behavior of the CC hydrogel. The shear strain-dependent modulus of the CC_1.5_ hydrogel is demonstrated in Figure 5A. When the dynamic shear strain changed from 0.1 to 1500%, the G′ values decreased correspondingly and the gel-to-sol transition occurred at ~1006% strain. It was worth noting that the G” values increased continuously as the strain varied from 100 to 500%, reflecting the ongoing transformation of gel to sol. Figure 5B shows the SAXS curves of the CC_1.5_ hydrogel under different strains. Broad peaks were detected in the curves at relatively low strains and then decreased in intensities as the strain increased. Figure 5C illustrates the possible changes in the network structure of the hydrogel as the strain increases. At low strains, the hydrogel network is probably maintained by hydrogen bonding, cation–π interactions, and Schiff base reactions or Michael addition. As the strain increases, the network structure of the hydrogel was gradually destroyed because of the rupture of hydrogen bonds.

### 3.4. Injectability and Self-Healing Properties of the CC Hydrogel

**Figure 6:**
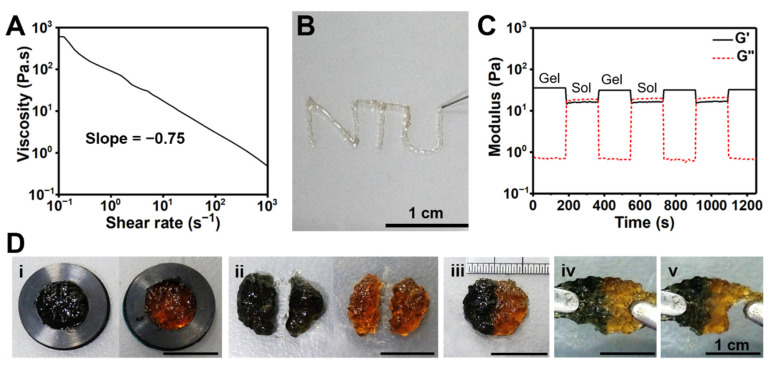
Injectability and self-healing properties of the CC hydrogel (solid content 1.5%). (**A**) The static shear viscosity versus shear rate of the hydrogel. (**B**) Injection of the hydrogel through a 30-gauge syringe needle. (**C**) Damage–healing cycles of the hydrogel evaluated at alternate 1% and 1100% dynamic strains and 1 Hz. (**D**) Gross observation for self-healing of the CC hydrogel: (i) the original hydrogel and the one colored with trypan blue were prepared in disc shape; (ii) each hydrogel was cut in half; (iii) two different pieces of hydrogels were contacted; (iv) the hydrogels were healed to an integral after 24 h; and (v) the hydrogel was pulled to failure but the breaking site was not at the healed site.

The shear-thinning property of the CC hydrogel is used to identify whether the hydrogel has good injectability or not. The static steady shear viscosity of the CC_1.5_ hydrogel is shown in Figure 6A. The slope of viscosity versus shear rate for the CC_1.5_ hydrogel was −0.75, indicating that the hydrogel had shear-thinning properties and good injectability. As illustrated in Figure 6B, the CC_1.5_ hydrogel could be injected through a 30-gauge syringe needle (160 µm internal diameter). The damage–healing cycles of the CC_1.5_ hydrogel were conducted at alternate dynamic strains between 1% and 1100% (Figure 6C). The CC_1.5_ hydrogel reached a sol-like state (G′′ > G′) at the higher strain (1100%) and recovered to a gel-like state (G′ > G′′) at the lower strain (1%). The CC_1.5_ hydrogel displayed good self-healing properties after repeated cycles. The macroscopic self-healing behavior of CC_1.5_ hydrogel is demonstrated in Figure 6D. The original hydrogel and the one colored with trypan blue were prepared in a disc shape and were cut into two pieces. After placing the two different pieces of hydrogels together for 24 h at room temperature, the hydrogels were healed to an integral. The healed hydrogel was stretched to failure, but the breaking site was different from the healed site.

### 3.5. Osmotic-Responsive Behavior and Adhesive Properties of the Hydrogels

**Figure 7:**
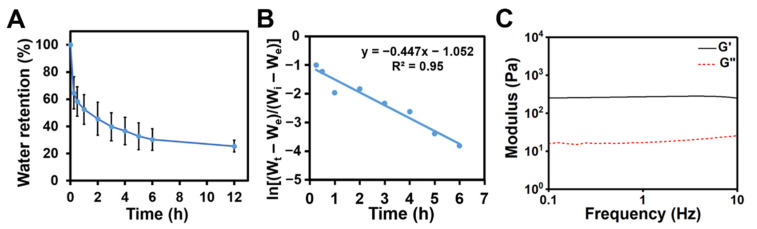
Osmotic-responsive properties of the CC hydrogel at 25 °C. (**A**) Deswelling behavior of the CC hydrogel in the physiological saline. (**B**) The deswelling kinetics shown as ln[(*W_t_* − *W_e_*)]/(*W_i_* − *W_e_*)] versus time *t*. (**C**) The rheological property of the deswollen CC hydrogel after immersion in saline for 12 h.

The CC_1.5_ hydrogel was immersed in physiological saline (0.9% NaCl solution) for a given time to evaluate the osmotic-responsive performance of the hydrogel. With the increased soaking time, the volume of the hydrogel gradually shrank due to the deswelling phenomenon of the hydrogel. The deswelling behavior of the hydrogel is shown in Figure 7A. The hydrogel exhibited rapid deswelling behavior when initially immersed in saline. The deswelling reached an equilibrium after 12 h. The equilibrated water retention of the hydrogel was about 25.5%. The deswelling kinetics were analyzed using Equation (2). The plot of ln[(*W_t_* − *W_e_*)/(*W_i_* − *W_e_*)] against a deswelling time *t* for the hydrogel was linear (Figure 7B). The deswelling rate constant *k* was found to be 0.45, suggesting that the deswelling process may be dominated by the first-ordered manner [30].

The moduli of the deswollen hydrogel are shown in Figure 7C. The storage modulus of the deswollen hydrogel at the 1 Hz frequency was about 270 Pa, which was significantly greater than the initial CC_1.5_ hydrogel (45.7 Pa). Meanwhile, the CC_1.5_ hydrogel immersed in culture medium also showed osmotic-responsive behavior (Figure S2, Supplementary Material). The hydrogel exhibited rapid deswelling behavior initially and then gradually reswelled after 15 min. The reswelling reached equilibrium after 12 h and the equilibrated water retention of the hydrogel was about 69.7%. Therefore, the CC hydrogel responded to osmotic changes in the environment.

**Figure 8:**
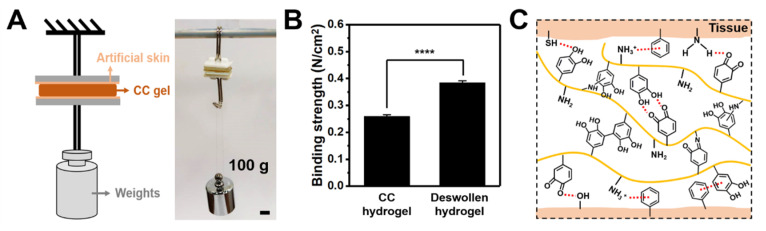
Adhesive properties of the CC hydrogel. (**A**) A weight-bearing equipment showing the binding between two pieces of artificial skins by the CC hydrogel. (**B**) The binding strength of the hydrogel and the deswollen one in saline for 12 h. **** represents *p* < 0.0001. (**C**) Illustration of the possible adhesive mechanism of the CC hydrogel with tissue.

The weight-bearing experiments were employed to evaluate the adhesive properties of the pristine CC_1.5_ hydrogel and the deswollen one in saline for 12 h (Figure 8A). The two glass slides in a contact area of 2 × 2 cm^2^ were attached with the commercial artificial skins and then glued together by the CC hydrogel. The combination of the CC_1.5_ hydrogel resisted 100 g of weight, while the deswollen hydrogel in saline endured over 150 g of weight. Figure 8B shows that the binding strength of the deswollen hydrogel (0.38 N/cm^2^) was greater than the initial CC_1.5_ hydrogel (0.26 N/cm^2^), indicating the better adhesiveness of the deswollen CC_1.5_ hydrogel. The possible adhesive mechanism of the CC hydrogel with tissue is shown in Figure 8C. The CC hydrogel can bind strongly to tissue surfaces possibly through the formation of π-π stacking, hydrogen bonding, and cation–π interactions.

### 3.6. Preparation and Characterization of the CC Scaffolds

**Figure 9:**
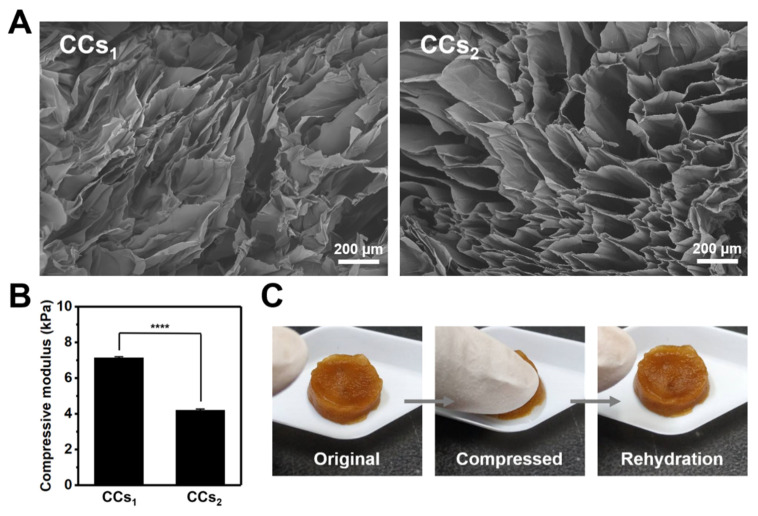
Properties of CCs_1_ (the lyophilized CC_1.5_ hydrogel) and CCs_2_ (the lyophilized CC_2.0_ hydrogel) scaffolds. (**A**) SEM images of the CCs_1_ and CCs_2_ scaffolds. (**B**) The compressive modulus of rehydrated CC scaffolds measured at a 1% compressive strain and 1 Hz frequency. **** represents *p* < 0.0001. (**C**) The macroscopic behavior of the CCs_2_ scaffold showing high compressibility.

The CC scaffolds were prepared by placing the CC hydrogels at −20 °C for 24 h followed by freeze-dried for 24 h. The lyophilized CC_1.5_ hydrogel was denoted as CCs_1_ scaffold and the lyophilized CC_2.0_ hydrogel was denoted as CCs_2_ scaffold. The porosity, swelling ratio, and compressive modulus of CC scaffolds are listed in Table 2. The SEM images of the CC scaffolds in Figure 9A illustrated that the CCs_1_ scaffold had a denser network, whereas the CCs_2_ scaffold had a continuous macroporous structure. The average pore size of the CCs_2_ scaffold was approximately 200 µm. The interconnected macroporous structure and high porosity (98%) of the CCs_2_ scaffold were similar to the case in cryogel. Figure 9B shows that the compressive modulus of the CCs_1_ scaffold (~7 kPa) was significantly larger than the CCs_2_ scaffold (~4 kPa), which may be associated with the denser network structure of the former scaffold and account for its insufficient resilience. Meanwhile, the interconnected macroporous structure of the CCs_2_ scaffold agreed with its good compressibility and swelling ratio (9656%). The hydrated CCs_2_ scaffold can resist compression and rapidly recover to the original shape after rehydration (Figure 9C and Video S1 (Supplementary Material)).

## 4. Discussion

Chitosan is an attractive naturally derived polymer with a wide range of biomedical applications, yet is often limited by the poor solubility at neutral pH due to inter-/intra-molecular hydrogen bonds [31]. After chemical modification, such as acylation and carboxylation, chitosan is endowed the water solubility [32]. In the current study, water-soluble catechol-grafted chitosan was successfully synthesized via EDC/NHS chemistry. The partial replacement of primary amines in chitosan with catechol groups may disrupt the inter-/intra-molecular hydrogen bonding, leading to increased solubility in aqueous solution at the neutral pH [33]. The solubility of CC in neutral solution mainly depends on the substitution degree of catechol to chitosan. Previous literature reported that the solubility of CC was less than 6 mg/mL (about 0.6 wt%) when the substitution degree of catechol was higher than 36% and the solubility may increase (~35 mg/mL) at pH 7.0 if the degree of substitution was lower than 15% [8]. Therefore, CC was prepared with a substitution degree of ~13% in the present study. However, the lyophilized CC was found to have low water solubility. The flow behavior of the CC after dialysis was directly examined. Rheological studies showed that the storage modulus (G′) was greater than the loss modulus (G′′) even at the low solid content (0.2 wt%), while both values were low and unstable, indicating that a colloidal suspension may have formed. During the freezing process of lyophilization, the distances between CC main chains may decrease as a result of ice crystal formation. The decreased chain distance may enhance the crosslinking reaction of catechol groups [34], resulting in poor solubility after being freeze-dried.

Because of the difficulty in dissolving the lyophilized CC, the CC hydrogels with solid contents greater than 0.5% were produced by rotary concentration in this study. Through the condensation that shortens the chain distance, the catechol groups on the CC backbone may react with the nearby amines or catechol groups more readily and enhance the catechol group-dominated crosslinking reaction kinetics to promote gelation [34]. The key mechanism of CC self-crosslinking to form the CC hydrogel is speculated to be Michael addition or Schiff base reactions accompanied by hydrogen bonding and cation–π interactions. Catechol is easily oxidized during the gelation process to form reactive catechol-quinone [35], which can undergo Michael addition or Schiff base reactions with adjacent amino groups in the chitosan backbone [21,36]. This process increases the cohesion of the hydrogel through covalent crosslinking [17]. In addition, the protonated amines on the CC polymer chains can institute cation–π interactions (either intramolecular or intermolecular) [37]. Cation–π interactions improve the cohesion of polymers rich in cationic and aromatic functional groups, which are the dominant mechanisms of molecular cohesion [16]. Furthermore, the formation of hydrogen bonds also contributes to the polymer cohesion [18]. CC hydrogels with a solid content of 1.5 wt% (CC_1.5_) and 2.0 wt% (CC_2.0_) reached an equilibrium state (with steady G′) in about two days. Moreover, there was no substantial difference in the G′ values between the two hydrogels (i.e., 45.7 Pa for CC_1.5_ and 52.8 Pa for CC_2.0_). The SEM images of the dried hydrogels revealed that the lyophilized CC_1.5_ hydrogel (i.e., CCs_1_ scaffold) had a relatively tight network structure, while the lyophilized CC_2.0_ hydrogel (i.e., CCs_2_ scaffold) showed macropores (~200 µm) similar to a cryogel [34]. The difference in the internal network structure may account for the G′ value which did not further increase from the CC_1.5_ hydrogel to the CC_2.0_ hydrogel due to a change in the gel network structure. Based on SAXS profiles, the structure change may be associated with regain of the larger-scale structure (>10 nm, or q < 0.01 Å^−1^ region).

The coherent SAXS technique, as a powerful instrument for the in situ structural investigation, has been widely utilized in hydrogel systems [28,38]. Our results demonstrated the structural stability of concentrated CC hydrogels. As the solid contents increased, the local chain distance in CC hydrogels may become closer and further arouse the self-assemble phenomenon by non-covalent interactions. The CC_1.5_ hydrogel was denoted as a critical group, above which the hydrogel started to have an obvious form factor deriving from spheroidal clusters at the q range ~0.02–0.04 Å^−1^. The scale of these clusters was estimated to be ~9 nm and the clusters only began to form above critical concentration, i.e., 1.5 wt%. These continuous clusters constituted a compact structure and led to a gradually formed hydrogel network with stable G′. Compared to the CC_1.5_ hydrogel, the scattering intensity of CC_2.0_ hydrogel slightly increased on a large scale (q < 0.01 Å^−1^), which may be caused by the increased solid contents. SAXS analyses revealed the increased density in the large-scale structure for CC_2.0_ hydrogels, which may explain the decreased pore sizes in SEM images as well as increased porosity (Table 2) of the CC_2.0_ hydrogel vs. the CC_1.5_ hydrogel. The slope of the curves did not change in CC_1.5_ and CC_2.0_ hydrogels in the high-q region, suggesting that the large-scale structures in both hydrogels were similar. Subsequently, the critical CC_1.5_ hydrogel was evaluated by the strain-dependent in situ SAXS experiments to verify the structural change in different dynamic shear strains. The tan δ was increased from 0.02 to 1 as the strain increased from 0.1 to 1006%. Such a change in tan δ denotes that the hydrogel undergoes a gel to sol transition. Meanwhile, a notable rise in the G′′ of the CC_1.5_ hydrogel happened in the strain ranging from 100% to 300%. This region (100 to 300%) may be correlated with the greatly reduced physical interactions, i.e., the hydrogen bonding, cation–π interactions, and Schiff base reactions or Michael addition. The breakage of physical interactions offers a pathway for energy dissipation in the strained CC_1.5_ hydrogel, as illustrated in Figure 5C.

Self-healing hydrogels which possess the ability to repair themselves after suffering the damage demonstrate the protracted lifetime [39]. The self-healing of CC hydrogels was mainly associated with the hydrogen bonding and Schiff base reactions between oxidized catechol–quinone groups and amino groups. Rheological experiments verified the self-healing ability of the CC hydrogel after repeated damage–healing cycles. Macroscopic experiments confirmed that the CC hydrogel healed to an integral within 24 h after being cut in half, and the breaking site was not at the healed site after being stretched. These results indicate that the CC hydrogel had good self-healing properties. The CC hydrogel also exhibited excellent shear-thinning properties, which is important for injectability. As was observed, the CC hydrogel was continuously injected through a very small needle (30-gauge, 160 μm inner diameter).

The swelling/deswelling behavior of a hydrogel in the ionic environment of physiological saline is in general related to the osmotic responsiveness. The CC hydrogel exhibited significant deswelling behavior after immersion in physiological saline for 12 h, accompanied by the volume shrinkage and modulus enhancement. The ionic environment of saline may induce the electrostatic shielding between chitosan chains and facilitate the physical crosslinking via hydrogen bonding in the gel [40]. As the crosslinking density increases, the denser network structures induce the volume shrinkage accompanied with water exclusion and increased strength [41]. In addition, salt enhanced the cation–π interactions, leading to the dehydration of the hydrogel due to the aggregation of polymer chains [42]. As gaseous oxygen plays an important role in the oxidation of catechol, further oxidation of catechol can occur during the dehydration process, which increases the local concentration of the hydrogel and enhances the mechanical strength [43]. On the other side, the CC hydrogel was immersed in glucose containing cell culture medium to contrast the osmotic-responsive behavior (Figure S2). Upon initial immersion in the medium, the CC hydrogel also displayed a rapid deswelling behavior, but then gradually reswelled unlike that in the saline. Hydrogel dehydration resulting from different osmotic pressures may contribute to the rapid deswelling of the hydrogel as that in the saline. Meanwhile, reswelling of the hydrogel may be triggered by interactions between small molecules such as amino acids/glucose and the CC main chains within the hydrogel. In the literature, the shrinking and swelling behavior of the hydrogel in the medium is determined by ionic interactions, which is associated with the osmotic pressure differences between the hydrogel and the external solutions [44]. The hydrogel can swell in the presence of an osmotic pressure caused by the difference in the mobile ion concentrations between the inner of the hydrogel and the outer solution [45].

Mussel-inspired hydrogel has been applied to prepare tissue adhesives due to the strong wet adhesion between various substrates in humid environments [46]. In the current study, CC hydrogels facilitated the adhesion and cohesion of catechol chemistry because of conjugation of catechol groups onto the chitosan that was rather non-adhesive in the non-conjugated state. The weight-bearing experiments (Figure 8A) showed that the catechol group was a key factor in generating adhesion. The strong adhesion of the hydrogel with artificial skin probably takes place through cation–π interactions, π-π stacking, and hydrogen bonding [47,48]. In addition, the adhesion of the CC hydrogel after immersion in saline was further enhanced, which was revealed by the binding strength shown in Figure 8B. Enhancing the cation–π interactions of the CC hydrogel in saline may contribute to the increase in cohesion and adhesion, based on the literature [49]. Such reinforced adhesion in physiological saline can favor for the in vivo applications as tissue adhesives or sealants for bleeding wounds [41].

Porous CC scaffolds can be further prepared by the lyophilization of CC hydrogels after freezing at −20 °C. SEM unveiled that the structure of the lyophilized CC_2.0_ hydrogel (i.e., CCs_2_ scaffold) was cryogel-like, which had a continuous macroporous structure (~200 µm) and high porosity (98%) (Table 2). The cryogel-like structure may be attributed to the increased crosslinking density, which leads to the irreversible formation of wall structures within the scaffold and results in a unique macroporous structure [34]. Moreover, the higher solid content may result in the stronger aggregation behavior of CC due to the increased chance of contact between the catechol groups. The aggregation may cause more heterogeneous structure, leading to the formation of a macroporous structure with larger porous size in CCs_2_. The CCs_2_ scaffold exhibited good compressibility and water absorption (9656%) due to the interconnected macroporous structure. The hydrated CCs_2_ scaffold squeezed water out after compression, and was able to quickly return to its original shape by taking the water back. Meanwhile, the lyophilized CC_1.5_ hydrogel (i.e., CCs_1_ scaffold) displayed lower compressibility, which was caused by its denser network structure. Compressive moduli of hydrated CC scaffolds verified the above results. The CCs_1_ scaffold had a higher compressive modulus (~7 kPa) than the CCs_2_ scaffold (~4 kPa). The CCs_1_ scaffold had larger stiffness, which also sacrificed its compressibility.

In summary, the catechol-grafted chitosan was successfully synthesized and the self-healing CC hydrogel was prepared through rotary concentration. The gelation behavior of the CC hydrogel was further explored by rheology and SAXS, which may require further investigation to confirm the mechanism. The CC hydrogel possessed good injectability, self-healing ability, and wet adhesion. In addition, the CC hydrogel exhibited osmotic responsiveness to saline. The CC hydrogel after immersion in saline enabled volume shrinkage and improvements in mechanical and adhesion properties. Moreover, the CC scaffold with excellent compressibility and water absorption was prepared by lyophilizing the hydrogel. Based on the above characteristics, the CC hydrogel and scaffold are potential materials with biomedical applications. The CC hydrogel can be developed as osmotic-responsive tissue adhesives or wound dressings, while the CC scaffold can be used as hemostatic sponges to stop bleeding or as a tissue engineering scaffold for regenerative medicine.

## 5. Conclusions

Catechol-grafted chitosan was synthesized and further concentrated to produce the self-healing CC hydrogels with different solid contents. The gelation mechanism of the self-healing hydrogel may be attributed to the synergistic effect of multiple reactions, including the formation of hydrogen bonding, cation–π interactions, Michael addition, or Schiff base reactions during the concentration process. The stability, self-healing ability, and good injectability of the CC hydrogel were verified by rheological studies. Coherent SAXS profiles and SEM images showed different network structures in CC hydrogels with diverse solid contents. The strain sweep experiments and in situ SAXS analyses confirmed the strain-dependent structure of the hydrogel. The hydrogel was osmotic-responsive to saline which enhanced the adhesive strength (0.38 N/cm^2^). Furthermore, lyophilizing the CC hydrogel produced high-water-absorbent (9656%) CC scaffolds with adequate durability and high compressibility. These findings show that the CC hydrogel and porous scaffolds developed in this study may be potential candidates for tissue adhesives, hemostatic materials, and tissue engineering scaffolds.

## Figures and Tables

**Table 1:** The storage modulus (G′) at 1 Hz and abbreviated names of CC hydrogels with different solid contents.

Hydrogels	Solid Contents (wt%)	Storage Modulus (G′, Pa)
CC_0.2_	0.2	0.3
CC_0.5_	0.5	0.4
CC_1.0_	1.0	20.6
CC_1.5_	1.5	45.7
CC_2.0_	2.0	52.8

**Table 2:** Basic properties of the CC scaffolds with different formulae *^a.^*

Scaffolds	Lyophilized Hydrogels	Porosity (%)	Swelling Ratio (%)	Compressive Modulus (kPa)
CCs_1_	CC_1.5_	96.6 ± 1.3	6646 ± 127	7.13 ± 0.07
CCs_2_	CC_2.0_	98.4 ± 0.9	9656 ± 93	4.19 ± 0.08

*^a^* The compressive modulus (dynamic state, 1% compressive strain, 1 Hz frequency, and 25 °C) was determined on hydrated CC scaffolds.

## Data Availability

Data sharing is not applicable to this article.

## Supplementary Materials

The following supporting information can be downloaded at: https://www.mdpi.com/article/10.3390/polym14214614/s1. Figure S1: The calibration curve of hydrocaffeic acid. Figure S2: Deswelling behavior of the CC hydrogel. Video S1: Compressibility and fast shape recovery of the CC scaffold.

## References

[1] Ojeda-Hernández D.D., Canales-Aguirre A.A., Matias-Guiu J., Gomez-Pinedo U., Mateos-Díaz J.C. (2020). Potential of chitosan and its derivatives for biomedical applications in the central nervous system. Front. Bioeng. Biotechnol..

[2] Qin Y., Li P. (2020). Antimicrobial chitosan conjugates: Current synthetic strategies and potential applications. Int. J. Mol. Sci..

[3] Xie W., Xu P., Liu Q. (2001). Antioxidant activity of water-soluble chitosan derivatives. Bioorganic Med. Chem. Lett..

[4] Nguyen N.T., Hoang D.Q., Nguyen N.D., Nguyen Q.H., Nguyen D.H. (2017). Preparation, characterization, and antioxidant activity of water-soluble oligochitosan. Green Process. Synth..

[5] Knight D.K., Shapka S.N., Amsden B.G. (2007). Structure, depolymerization, and cytocompatibility evaluation of glycol chitosan. J. Biomed. Mater. Res. A..

[6] Sashiwa H., Kawasaki N., Nakayama A., Muraki E., Yamamoto N., Aiba S.-i. (2002). Chemical modification of chitosan. 14: Synthesis of water-soluble chitosan derivatives by simple acetylation. Biomacromolecules.

[7] Chen X.-G., Park H.-J. (2003). Chemical characteristics of O-carboxymethyl chitosans related to the preparation conditions. Carbohydr. Polym..

[8] Kim K., Ryu J.H., Lee D.Y., Lee H. (2013). Bio-inspired catechol conjugation converts water-insoluble chitosan into a highly water-soluble, adhesive chitosan derivative for hydrogels and LbL assembly. Biomater. Sci..

[9] Rasool A., Ata S., Islam A. (2019). Stimuli responsive biopolymer (chitosan) based blend hydrogels for wound healing application. Carbohydr. Polym..

[10] Annabi N., Rana D., Sani E.S., Portillo-Lara R., Gifford J.L., Fares M.M., Mithieux S.M., Weiss A.S. (2017). Engineering a sprayable and elastic hydrogel adhesive with antimicrobial properties for wound healing. Biomaterials.

[11] Pellá M.C., Lima-Tenório M.K., Tenório-Neto E.T., Guilherme M.R., Muniz E.C., Rubira A.F. (2018). Chitosan-based hydrogels: From preparation to biomedical applications. Carbohydr. Polym..

[12] Roberts G.A., Taylor K.E. (1989). Chitosan gels, 3. The formation of gels by reaction of chitosan with glutaraldehyde. Die Makromol. Chem. Macromol. Chem. Phys..

[13] Liu Y., Lin S.-H., Chuang W.-T., Dai N.-T., Hsu S.-h. (2022). Biomimetic strain-stiffening in chitosan self-healing hydrogels. ACS Appl. Mater. Interfaces.

[14] Park E., Lee J., Huh K.M., Lee S.H., Lee H. (2019). Toxicity-Attenuated Glycol Chitosan Adhesive Inspired by Mussel Adhesion Mechanisms. Adv. Healthc. Mater..

[15] Ryu J.H., Hong S., Lee H. (2015). Bio-inspired adhesive catechol-conjugated chitosan for biomedical applications: A mini review. Acta Biomater..

[16] Gebbie M.A., Wei W., Schrader A.M., Cristiani T.R., Dobbs H.A., Idso M., Chmelka B.F., Waite J.H., Israelachvili J.N. (2017). Tuning underwater adhesion with cation–π interactions. Nat. Chem..

[17] Saiz-Poseu J., Mancebo-Aracil J., Nador F., Busqué F., Ruiz-Molina D. (2019). The chemistry behind catechol-based adhesion. Angew. Chem. Int. Ed..

[18] Zhang W., Wang R., Sun Z., Zhu X., Zhao Q., Zhang T., Cholewinski A., Yang F.K., Zhao B., Pinnaratip R. (2020). Catechol-functionalized hydrogels: Biomimetic design, adhesion mechanism, and biomedical applications. Chem. Soc. Rev..

[19] Yang J., Stuart M.A.C., Kamperman M. (2014). Jack of all trades: Versatile catechol crosslinking mechanisms. Chem. Soc. Rev..

[20] Xu L.Q., Yang W.J., Neoh K.-G., Kang E.-T., Fu G.D. (2010). Dopamine-induced reduction and functionalization of graphene oxide nanosheets. Macromolecules.

[21] Wu J., Zhang L., Wang Y., Long Y., Gao H., Zhang X., Zhao N., Cai Y., Xu J. (2011). Mussel-inspired chemistry for robust and surface-modifiable multilayer films. Langmuir.

[22] Lee H., Lee K.D., Pyo K.B., Park S.Y., Lee H. (2010). Catechol-grafted poly (ethylene glycol) for PEGylation on versatile substrates. Langmuir.

[23] Hong S., Yang K., Kang B., Lee C., Song I.T., Byun E., Park K.I., Cho S.W., Lee H. (2013). Hyaluronic acid catechol: A biopolymer exhibiting a pH-dependent adhesive or cohesive property for human neural stem cell engineering. Adv. Funct. Mater..

[24] Lee C., Shin J., Lee J.S., Byun E., Ryu J.H., Um S.H., Kim D.-I., Lee H., Cho S.-W. (2013). Bioinspired, calcium-free alginate hydrogels with tunable physical and mechanical properties and improved biocompatibility. Biomacromolecules.

[25] Ryu J.H., Lee Y., Kong W.H., Kim T.G., Park T.G., Lee H. (2011). Catechol-functionalized chitosan/pluronic hydrogels for tissue adhesives and hemostatic materials. Biomacromolecules.

[26] Xu J., Strandman S., Zhu J.X., Barralet J., Cerruti M. (2015). Genipin-crosslinked catechol-chitosan mucoadhesive hydrogels for buccal drug delivery. Biomaterials.

[27] Yavvari P.S., Srivastava A. (2015). Robust, self-healing hydrogels synthesised from catechol rich polymers. J. Mater. Chem. B.

[28] Lin S.-H., Papadakis C.M., Kang J.-J., Lin J.-M., Hsu S.-h. (2021). Injectable phenolic-chitosan self-healing hydrogel with hierarchical micelle architectures and fast adhesiveness. Chem. Mater..

[29] Serizawa T., Wakita K., Akashi M. (2002). Rapid deswelling of porous poly (*N*-isopropylacrylamide) hydrogels prepared by incorporation of silica particles. Macromolecules.

[30] Ma J., Zhang L., Fan B., Xu Y., Liang B. (2008). A novel sodium carboxymethylcellulose/poly (*N*-isopropylacrylamide)/Clay semi-IPN nanocomposite hydrogel with improved response rate and mechanical properties. J. Polym. Sci. Part B Polym. Phys..

[31] Aljawish A., Chevalot I., Jasniewski J., Scher J., Muniglia L. (2015). Enzymatic synthesis of chitosan derivatives and their potential applications. J. Mol. Catal. B Enzym..

[32] Nishimura S., Kohgo O., Kurita K., Kuzuhara H. (1991). Chemospecific manipulations of a rigid polysaccharide: Syntheses of novel chitosan derivatives with excellent solubility in common organic solvents by regioselective chemical modifications. Macromolecules.

[33] Liu Y., Wong C.-W., Chang S.-W., Hsu S.-h. (2021). An injectable, self-healing phenol-functionalized chitosan hydrogel with fast gelling property and visible light-crosslinking capability for 3D printing. Acta Biomater..

[34] Park E., Ryu J.H., Lee D., Lee H. (2021). Freeze–Thawing-Induced Macroporous Catechol Hydrogels with Shape Recovery and Sponge-like Properties. ACS Biomater. Sci. Eng..

[35] Waite J.H. (1990). The phylogeny and chemical diversity of quinone-tanned glues and varnishes. Comp. Biochem. Physiol. Part B Comp. Biochem..

[36] Yang J., Saggiomo V., Velders A.H., Cohen Stuart M.A., Kamperman M. (2016). Reaction pathways in catechol/primary amine mixtures: A window on crosslinking chemistry. PLoS ONE.

[37] Ma J.C., Dougherty D.A. (1997). The cation−π interaction. Chem. Rev..

[38] Lin Y.-J., Chuang W.-T., Hsu S.-h. (2019). Gelation mechanism and structural dynamics of chitosan self-healing hydrogels by in situ SAXS and coherent X-ray scattering. ACS Macro Lett..

[39] He J., Shi M., Liang Y., Guo B. (2020). Conductive adhesive self-healing nanocomposite hydrogel wound dressing for photothermal therapy of infected full-thickness skin wounds. Chem. Eng. J..

[40] Yang Y., Wang X., Yang F., Shen H., Wu D. (2016). A universal soaking strategy to convert composite hydrogels into extremely tough and rapidly recoverable double-network hydrogels. Adv. Mater..

[41] Fang W., Yang L., Hong L., Hu Q. (2021). A chitosan hydrogel sealant with self-contractile characteristic: From rapid and long-term hemorrhage control to wound closure and repair. Carbohydr. Polym..

[42] Kim S., Yoo H.Y., Huang J., Lee Y., Park S., Park Y., Jin S., Jung Y.M., Zeng H., Hwang D.S. (2017). Salt triggers the simple coacervation of an underwater adhesive when cations meet aromatic π electrons in seawater. ACS Nano.

[43] Shin M., Park S.-G., Oh B.-C., Kim K., Jo S., Lee M.S., Oh S.S., Hong S.-H., Shin E.-C., Kim K.-S. (2017). Complete prevention of blood loss with self-sealing haemostatic needles. Nat. Mater..

[44] Holtz J.H., Asher S.A. (1997). Polymerized colloidal crystal hydrogel films as intelligent chemical sensing materials. Nature.

[45] Chang C., He M., Zhou J., Zhang L. (2011). Swelling behaviors of pH-and salt-responsive cellulose-based hydrogels. Macromolecules.

[46] Rahimnejad M., Zhong W. (2017). Mussel-inspired hydrogel tissue adhesives for wound closure. Rsc Adv..

[47] Yang B., Ayyadurai N., Yun H., Choi Y.S., Hwang B.H., Huang J., Lu Q., Zeng H., Cha H.J. (2014). In Vivo residue-specific dopa-incorporated engineered mussel bioglue with enhanced adhesion and water resistance. Angew. Chem..

[48] Choi Y.C., Choi J.S., Jung Y.J., Cho Y.W. (2014). Human gelatin tissue-adhesive hydrogels prepared by enzyme-mediated biosynthesis of DOPA and Fe^3+^ ion crosslinking. J. Mater. Chem. B.

[49] Kim S., Faghihnejad A., Lee Y., Jho Y., Zeng H., Hwang D.S. (2015). Cation–π interaction in DOPA-deficient mussel adhesive protein mfp-1. J. Mater. Chem. B.

