# Supplementary material for: Gelation and the Self-Healing Behavior of the Chitosan–Catechol Hydrogel
Source: Polymers (Basel). 2022 Oct 30;14(21):4614. doi: 10.3390/polym14214614 (PMC9653763; doi:10.3390/polym14214614)
Supplement: Supplementary file 1 [file polymers-1985396 Supplementary peer review.pdf › polymers-1985396 Supplementary peer review.pdf]

polymers

MDPI

Supplementary Material: Gelation and Self-Healing Behavior of Chitosan-Catechol Hydrogel

Yu-Ting Lan, Qian-Pu Cheng, Junpeng Xu, Shih-Ho Lin, Jhih-Min Lin, Shan-hui Hsu

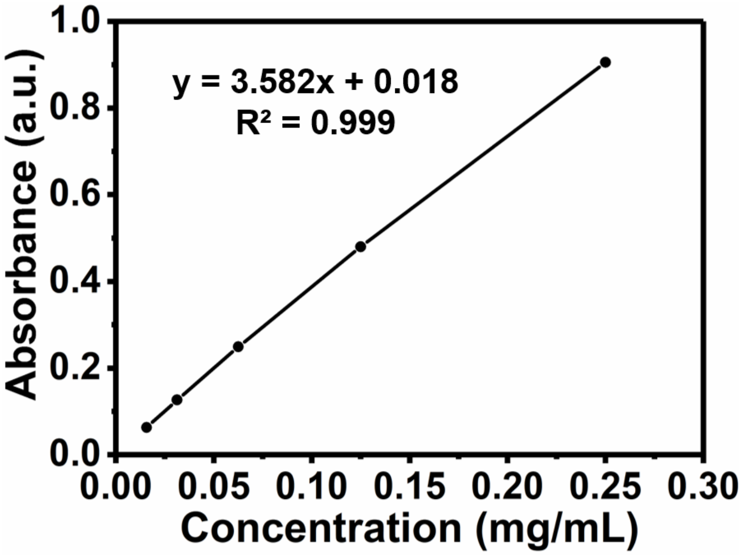

Figure S1. A calibration curve generated from the standard hydrocaffeic acid solutions at the absorption intensity of 280 nm. The coefficient of determination (R^2) was 0.999.

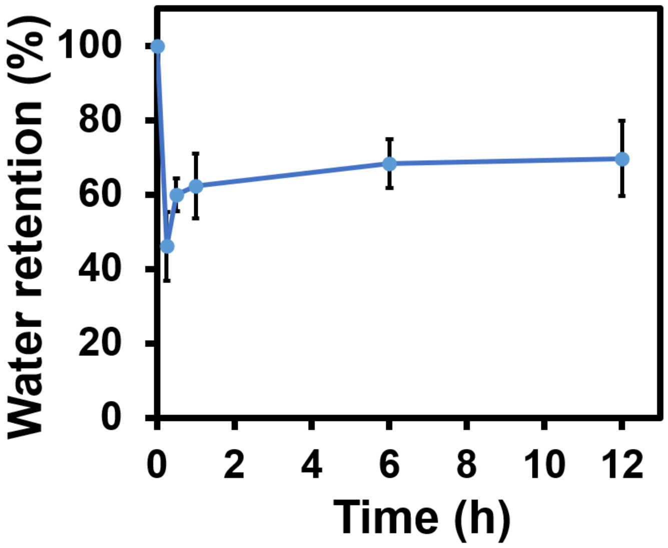

Figure S2. Deswelling behavior of the CC hydrogel in culture medium (low-glucose DMEM) at 25 °C. Compared to Figure 7, reswelling occurred after 15 min and the equilibrated water content was about 69.7%.

Video S1. Compressibility and fast shape recovery. The CC scaffold (CCs_2 as an example) can endure compression and expel water once being pressed, and can quickly recover to the original shape and take the water back after release of the compression force.
